# Dr. Sluman C. Crittenden

**Published:** 1914-06

**Authors:** 


Dr. Sluman C. Crittenden, Philadelphia College of Medicine and Surgery, 1856; died in Detroit, April 28, aged 79. He was a surgeon of volunteers during the Civil War. He had practiced in Buffalo, Rochester and Batavia.

